# Role of mitochondrial translation in remodeling of energy metabolism in ER/PR(+) breast cancer

**DOI:** 10.3389/fonc.2022.897207

**Published:** 2022-08-30

**Authors:** Emine C. Koc, Fatih C. Koc, Funda Kartal, Maria Tirona, Hasan Koc

**Affiliations:** ^1^Department of Biomedical Sciences, Joan C. Edwards School of Medicine, Marshall University, Huntington, WV, United States; ^2^Department of Medical Oncology, Joan C. Edwards School of Medicine, Marshall University, Huntington, WV, United States; ^3^Department of Pharmaceutical Science, School of Pharmacy, Marshall University, Huntington, WV, United States

**Keywords:** mitochondrial translation, mitochondrial ribosomal proteins (MRPs), oxidative phosphorylation (OXPHOS), breast cancer, ER/PR(+), luminal A

## Abstract

Remodeling of mitochondrial energy metabolism is essential for the survival of tumor cells in limited nutrient availability and hypoxic conditions. Defects in oxidative phosphorylation (OXPHOS) and mitochondrial biogenesis also cause a switch in energy metabolism from oxidative to aerobic glycolysis contributing to the tumor heterogeneity in cancer. Specifically, the aberrant expressions of mitochondrial translation components such as ribosomal proteins (MRPs) and translation factors have been increasingly associated with many different cancers including breast cancer. The mitochondrial translation is responsible for the synthesis 13 of mitochondrial-encoded OXPHOS subunits of complexes. In this study, we investigated the contribution of mitochondrial translation in the remodeling of oxidative energy metabolism through altered expression of OXPHOS subunits in 26 ER/PR(+) breast tumors. We observed a significant correlation between the changes in the expression of mitochondrial translation-related proteins and OXPHOS subunits in the majority of the ER/PR(+) breast tumors and breast cancer cell lines. The reduced expression of OXPHOS and mitochondrial translation components also correlated well with the changes in epithelial-mesenchymal transition (EMT) markers, E-cadherin (CHD1), and vimentin (VIM) in the ER/PR(+) tumor biopsies. Data mining analysis of the Clinical Proteomic Tumor Analysis Consortium (CPTAC) breast cancer proteome further supported the correlation between the reduced OXPHOS subunit expression and increased EMT and metastatic marker expression in the majority of the ER/PR(+) tumors. Therefore, understanding the role of MRPs in the remodeling of energy metabolism will be essential in the characterization of heterogeneity at the molecular level and serve as diagnostic and prognostic markers in breast cancer.

## Introduction

Breast cancer is one of the common causes of cancer-related deaths in the world due to high rates of invasiveness, metastasis, and recurrence. Since the recognition of energy metabolism as one of the major hallmarks of cancer, many studies revealed the contribution of oxidative phosphorylation (OXPHOS) and mitochondrial metabolites to the heterogeneity of tumor cell metabolism in cancer (1–5). OXPHOS complexes, I-V, consists of mitochondrial and nuclear-encoded subunits localized to the inner mitochondrial membrane. Mitochondrial genome encodes for the 13 mRNAs, two rRNAs (12S and 16S), and 22 tRNAs to support OXPHOS in humans. These 13 mitochondrial (mt)-encoded essential subunits form the integral core of the OXPHOS complexes I, III, IV, and V (ATP synthase). mtDNA mutations, low copy numbers, and reduced mitochondrial transcript levels are associated with increased metastasis and poor prognosis in breast cancer (6–10). A subset of genetic variations in complex I and IV genes, MT-ND2 and MT-COII, has recently been related to breast cancer risk of European Americans in 2,723 breast cancer cases and 3,260 controls from a Multiethnic Cohort Study (11).

In addition to the changes in mt-encoded genes affecting OXPHOS capacity, nuclear-encoded factors supporting biogenesis of OXPHOS subunits such as mitochondrial transcription and translation machineries are also implicated in remodeling energy metabolism (12–17). Mitochondrial translation machinery is composed of 55S ribosomes and translation factors and its components are completely different from their cytosolic counterparts (18–24). In the initial proteomics studies of 55S ribosomes, we identified over 80 mitochondrial ribosomal proteins (MRPs) which are solely responsible for the synthesis of 13 essential OXPHOS subunits (20, 21, 25–27). Some MRPs have acquired additional roles in health and various diseases (28–31). For example, two previously identified pro-apoptotic MRPs, DAP3 and PDCD9 (also known as MRPS29 and MRPS30), are the *bona fide* components of the mitochondrial ribosome involved in apoptosis (32–38).

Altered expression of MRP genes is mostly detected in various carcinomas, and their possible roles in mitochondrial translation have been elegantly and systematically reviewed in several publications (30, 31, 39). Studies from multiple laboratories have shown the remodeling of the tumor metabolism by changes in expression of MRP transcripts while driving heterogeneity, invasive capabilities, and recurrence in breast cancer (40–47). In agreement with these reports, reduced DAP3 expression levels have been associated with local recurrence, distant metastasis, and mortality in breast tumors (48–50). A single nucleotide polymorphism (SNP) in chromosome 5p12 breast cancer susceptibility locus affects MRPS30 expression in estrogen receptor positive (ER(+)) tumors (43, 51–54). Long noncoding (LncRNA) MRPS30-divergent transcript (DT) is overexpressed in breast carcinoma and its knockdown significantly reduced cell proliferation and invasion in breast cancer cell lines (55). In two independent studies, overexpression of the MRPL13 gene is proposed as a possible prognostic biomarker and correlated with unfavorable clinical outcomes of breast cancer patients (56, 57). Similarly, the overexpression of the MRPS6 and MRPS23 genes affected tumorigenic cellular processes in breast cancer and their knockdown decreased proliferation, expression of select mesenchymal marker and increased expression of tumor suppressor genes (58–60). On the other hand, the reduced stability of MRPS23 by its methylation at arginine R21 has been shown to promote breast cancer metastasis through inhibiting OXPHOS subunit expression (61).

As summarized above, the altered gene expression for MRPs such as, DAP3, MRPS6, MRPS18A, MRPS18B, and MRPS23 has been detected in highly proliferative and aggressive forms of breast cancers (40, 45, 46, 48, 50, 60); however, their steady-state protein expressions have not been studied in relation to neither OXPHOS subunit nor the mitochondrial translation component expressions in the majority of these studies. Quantitative analyses of protein expression in breast cancer and its subtypes in an unbiased manner have been greatly facilitated by mass spectrometry (MS)-based proteogenomic studies (62–65).

In this study, we have used 26 ER/PR(+) biopsies from breast cancer patients and shown that the altered MRP expression was correlated with reduced mitochondrial translation and OXPHOS subunit expression. The mining of the breast cancer proteomics data published by the Clinical Proteomic Tumor Analysis Consortium (CPTAC) also supported our findings on the heterogeneity of OXPHOS subunit and MRP expression in breast cancer. These observations suggest that defects in mitochondrial translation components play an important role in the remodeling of the energy metabolism by altering OXPHOS subunit expression, and possibly, mitochondrial apoptotic pathways in ER/PR(+) breast cancer. The alterations in mitochondrial translation and its components could serve as diagnostic markers in determining heterogeneity of ER/PR(+) subtypes and predicting disease prognosis.

## Materials and methods

### Breast tissue biopsies

Twenty-six treatment-naïve **de-identified** ER/PR(+) tumor (invasive ductal carcinoma) and normal breast tissue biopsies were removed by surgical excision from patients treated at the Marshall University Edwards Comprehensive Cancer Center, Huntington, WV. Tumor characteristics of biopsy samples are given in **Table S1**. Breast cancer subtypes were determined by immunohistochemistry, immunofluorescence, and fluorescence *in situ* hybridization techniques by the Edwards Comprehensive Cancer. Tissue protein lysates were prepared by resuspension and sonication of biopsies in RIPA buffer containing 1% SDS and NP40. Protein concentration was determined by the bicinchoninic acid (BCA) assay (Pierce, Rockford, USA).

### Cell culture and pulse labeling

Human breast cell lines MCF10A, MCF7, and MDA-MB-231 cell lines were purchased from the American Type Culture Collection (ATCC). The MCF 10A cell line was cultured in Dulbecco’s Modified Eagle Medium/Nutrient Mixture F-12 (DMEM/F12 (1:1)) as recommended by ATCC. Monolayer cultures of MCF7 and MDA-MB-231 cell lines were maintained in DMEM (HyClone, Thermo-Scientific, Waltham, MA) containing 10% fetal bovine serum (FBS) (Rocky Mountain Biologicals, Missoula, MT), 4 mM glutamine, 1 mM pyruvate, and 1% penicillin/streptomycin (Corning Cellgro, Manassas, VA). The cells were grown in a humidified incubator at 37°C with 5% CO_2_.

The expression of the 13 mt-encoded subunits of OXPHOS complexes was determined by ^35^S-Met pulse labeling described previously (66). Pulse labeling experiments were performed with breast cancer cell lines grown to 60-70% confluency. After arresting cytosolic protein synthesis by emetine, cells were incubated in 0.2 mCi/mL of [^35^S]-methionine (Perkin Elmer) containing media for 2h. Cells were lysed in RIPA buffer supplemented with protease and phosphatase inhibitors (Sigma-Aldrich). Whole cell lysates (30 μg) were electrophoresed through 13% SDS-PAGE. The gels were dried on 3MM chromatography paper (Whatman), and the signal intensities of the bands were quantified by ImageJ.

### Immunoblotting analyses

Tissue lysates obtained from biopsies and cell lines were either diluted further or lysed in RIPA buffer containing 50 mM Tris-HCl (pH 7.6), 150 mM NaCl, 1 mM EDTA, 1 mM EGTA, 1% NP40, 0.1% SDS, 0.5% deoxycholate, and protease and phosphatase inhibitor cocktails. Protein concentrations were determined using BCA assays (Pierce, Rockford, USA). The protein lysates (usually 15-30 µg) were then separated on 12% SDS-PAGE, transferred to nitrocellulose membranes (Amersham, GE Healthcare, Pittsburg, PA), stained with Ponceau S to ensure equal protein loading. The antibodies were commercially obtained as follows: Human OXPHOS antibody cocktail and SDHA from Abcam (Eugene, OR); MRPS30, MRPL11, and MRPS18B antibodies from Sigma Aldrich (St. Louis, MO); DAP3 (or MRPS29) antibody from BD Biosciences (San Jose, CA); mitochondrial Citrate synthase (CS), DAR2, TSFM, TUFM and TFAM; antibodies from Santa Cruz (Dallas, TX); and the loading control antibody GAPDH from Fitzgerald (Acton, MA). The secondary anti-rabbit and mouse HRP-conjugate antibodies from (Pierce, Rockford, USA). The protein immunoreactivity was detected using the ECL Western blotting kit from Amersham (GE Healthcare, UK) and the membranes were developed per the manufacturer’s protocol. Immunoblotting signal intensities were quantified by ImageJ (67) and UN-Scan-It (Silk Scientific, Inc, Orem, UT) and normalized to Ponceau S stained membranes. Relative protein expression detected by immunoblotting analyses was normalized to protein loading adjusted by Ponceau S staining and the average of protein expression detected by the same antibody in all biopsies. Normalizing the expression of a protein in each tumor biopsy to the average of the expression of the same protein in tumor biopsies was also used in proteomics studies of breast cancer by CPTAC (65). Due to the differences in breast tumor and normal tissue albumin expression, relative protein expression was only compared in ER/PR(+) tumor biopsies to prevent unequal protein loading (**Supplementary Figures 1, 2**).

### Mitochondrial complex IV activity assays

Breast tissue biopsies and breast cancer cell lines were homogenized by sonication in Complex IV assay buffer (10 mM Tris-HCl, pH 7.0, 120 mM KCl, 250 mM sucrose, 1 mM n-Dodecyl-β-D maltoside). Protein concentration was determined by BCA assay. Complex IV activity was measured kinetically by oxidation of reduced ferrocytochrome c at 550 nm as previously described (68). Specific activities were calculated from the linear region of the kinetic measurements and normalized to the protein amounts using the Ponceau S staining (**Supplementary Figure 3**). The data are expressed as the mean ± SD of at least three independent experiments of triplicates.

### Quantitative reverse transcription polymerase chain reaction

Total RNA was extracted from breast tumor biopsies in the presence of TRIzol (Invitrogen, Carlsbad, CA) and converted to cDNA with the High-Capacity cDNA reverse transcription kit using random primers (Applied Biosystems, Inc., Foster City, CA). Quantitative real-time PCR (qRT-PCR) was carried out using the PowerUp SYBR green master mix (Applied Biosystems, Inc.), and samples were run on an Applied Biosystems Step One Plus instrument. The relative expression values were calculated using the ΔΔCt method for both biological and technical replicates (33). Relative mRNA expression levels were calculated using GAPDH as endogenous control and MCF10A values as calibrator. The following forward and reverse primers were used for quantitative real-time PCR.

GAPDH5' GTCTTCACCACCATGGAGAAGG 3' (FW)
5' ATGAGGTCCACCACCCTGTTGC 3' (REV)
ND65' CTCACCAAGACCTCAACCCC 3' (FW)
5' ATTGTTAGCGGTGTGGTCGG 3' (REV)
COI5' TTCGCCGACCGTTGACTATT 3' (FW)
5' GGGGGCACCGATTATTAGGG 3'(REV)
DAP35' AATCCCACTCAGTCAGAGCC 3' (FW)
5' CCAGTGGATGGAGTTGCCTT 3'(REV)
MRPL115' AAGCAGAGGGGGTTAGTGGT 3' (FW)
5' TGGGGGTTGTCTAGGATGGT 3' (REV)
MRPS18B 5' TCCTGACGTTACATTGTCCCC 3' (FW)
5' AGTCAGAGCCACCAGGTACA 3'(REV)

### Statistical analysis

Statistical and graphical analyses were performed using Excel and GraphPad Prism 9.3. All the values were in triplicates wherever possible and expressed as mean ± SD, unless otherwise described.

## Results and discussion

### Heterogeneity of mitochondrial energy metabolism in ER/PR(+) breast cancer

Increased energy metabolism, specifically OXPHOS, is implicated in the progression and metastasis of breast tumors and is suggested to cause resistance to endocrine treatment (47, 69). Studies revealing the heterogeneity of OXPHOS and the underlying factors can be essential to develop alternative treatment options and prognostic markers for ER/PR(+) breast cancer. To evaluate the modulation of mitochondrial energy metabolism and biogenesis, we obtained 26 treatment-naïve ER/PR(+) invasive ductal carcinoma biopsies. The tumor characteristics and stages of invasive ductal carcinoma biopsies are given in the **Table S1** and the majority of these biopsies were classified as ER/PR (+) subtype.

One of the convenient and effective ways to determine the changes in expression of proteins involved in OXPHOS is to detect steady-state expression of OXPHOS subunits by immunoblotting using an antibody cocktail that recognizes at least one subunit from each complex. The cocktail is a mixture of five antibodies generated against four of the nuclear-encoded subunits of complex V (ATP5A1), III (UQCRC2), II (SDHB), and I (NDUFB8) and a mt-encoded subunit of complex IV (MT-COII). We first compared OXPHOS subunit expression using equal protein loading in tumor and adjacent normal tissue biopsies for each patient. Due to the high albumin content detected by the Ponceau S staining in normal tissue biopsies, equal protein loading was not sufficient to demonstrate the changes in OXPHOS subunit expression in tumor biopsies relative to the normal tissue lysates. Clearly, the OXPHOS subunit expression was at least 2-3-fold higher in tumor biopsies (**Supplementary Figure 1**). This observation supports the increase in epithelial cell content in breast tumors and makes the interpretation of the increased mitochondrial energy metabolism in breast tumors relative to normal tissue more challenging.

**Figure 1:**
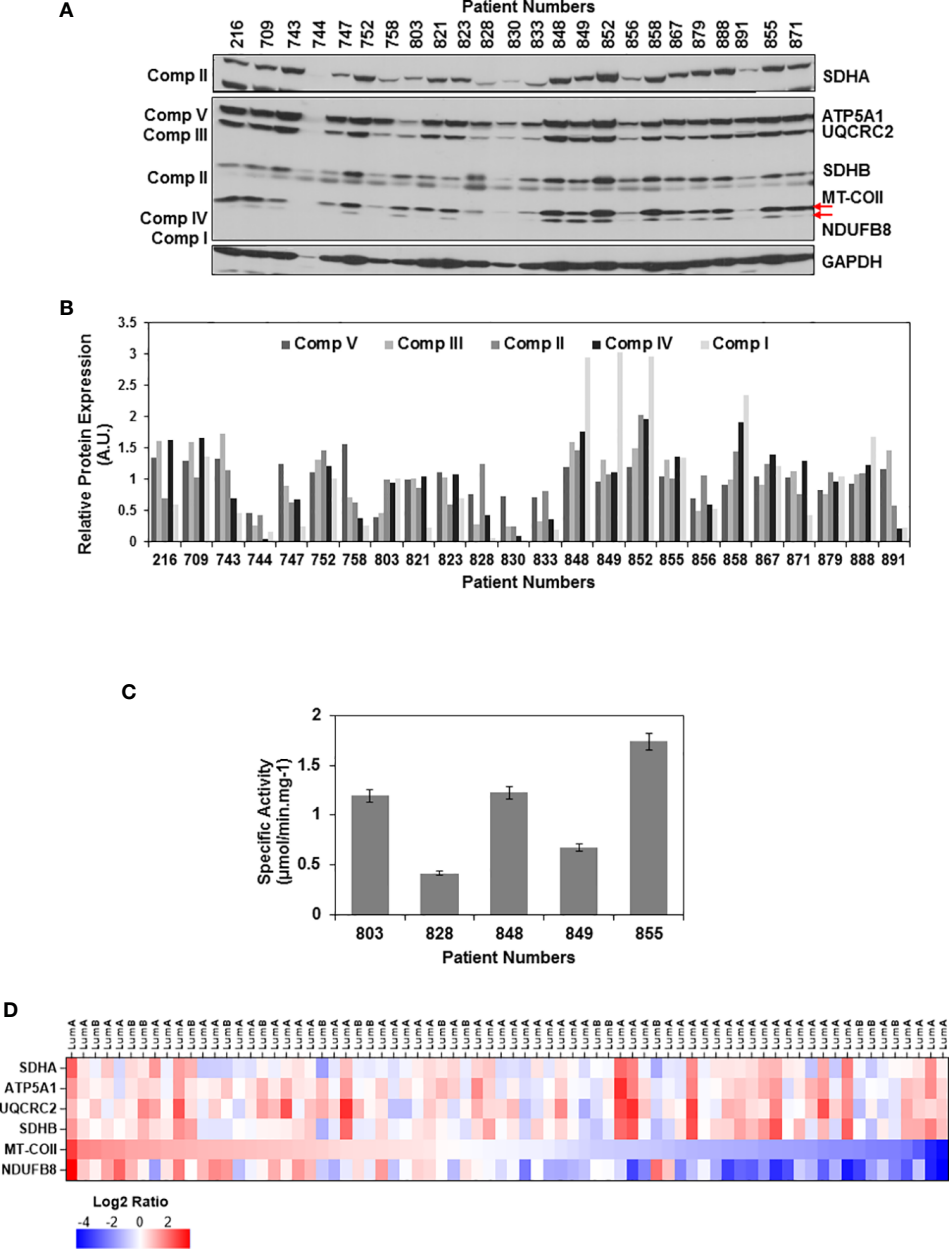
OXPHOS subunit expression heterogeneity in ER/PR(+) breast cancer. **(A)** The expression of OXPHOS subunits, including ATP5A1 (Complex V), UQCRC2 (Complex III), SDHA and SDHB (Complex II), MT-COII (Complex IV), and NDUFB8 (Complex I),were detected by immunoblotting in ER/PR(+) invasive ductal carcinoma biopsy lysates. Approximately, 20 μg of protein lysates obtained from tumor tissues was separated by 12% SDS-PAGE, and equal protein loading was evaluated by GAPDH antibody and Ponceau S staining (see **Figure S2**). **(B)** Quantitation of OXPHOS subunit expression in patient tumor biopsies by normalizing average signal intensities for each antibody to protein loading. AU; arbitrary units. **(C)** Complex IV specific activity was determined by measuring the rate of cytochrome c oxidation at 550 nm using equal amounts (~20 μg) of biopsy lysates selected from patients expressing high, medium, and low levels of MT-COII as shown in panel A. Results represent the mean ± SD of at least three experiments. **(D)** Heatmap representation of log2 ratios of OXPHOS subunit expression in luminal A and B subtypes of breast cancer detected by MS-based proteomics published by Krug et al. (65) (**Table S2**). Only the ER/PR(+) luminal A and B subtypes analyzed by the CPTAC used in the figure. The expression of OXPHOS subunits is ranked from high (red) to low (blue) MT-COII log2 ratios.

To evaluate the changes in mitochondrial energy metabolism in tumor biopsies, equal amounts of tumor lysates were separated on 12% SDS-PAGE and the same membrane blot was probed with OXPHOS, SDHA, and GAPDH antibodies (**Figure 1A** and **Supplementary Figure 2**). Relative expression of each OXPHOS subunit was quantified by normalizing protein loading to Ponceau S staining and the average signal detected by the corresponding antibody in all the tissues. Using this analysis, we were able to quantify OXPHOS subunit expression in ER/PR(+) tumor biopsies (**Figure 1B**). The change in expression of complex I and IV subunits, NDUFS8 and MT-COII, respectively, were more drastic than that of complex V, III, and II subunits (shown by arrows in **Figure 1A**). Although the NDUFB8 is a nuclear-encoded complex I subunit, its expression is highly sensitive to the reduced expression of seven mt-encoded complex I subunits which are shown to be critical for defining an aggressive phenotype in breast cancer (70).

The changes in MT-COII expression imply a defect in mitochondrial biogenesis as it is one of the three mt-encoded subunits of complex IV. To correlate the changes in MT-COII protein expression to complex IV activity, assays were performed using biopsies chosen from groups expressing varying steady-state levels of MT-COII (**Figure 1A**). Here, the complex IV activity assay serves as a measure of defects caused by MT-COI, MT-COII, and MT-COIII and other nuclear-encoded subunits of complex IV. The complex IV subunit expression and activity is determined to be the rate-controlling step in breast cancer (71). Not to our surprise, we also observed a direct correlation between the subunit expression (shown in **Figure 1A**) and the complex IV activities when biopsy samples from the same patient were compared (**Figure 1C**). Hence, the decrease detected in MT-COII expression is directly proportional to complex IV activities in these biopsies. Taken together, these observations suggest that the ER/PR(+) breast cancer can be further sub-classified into tumors with high, medium, and low OXPHOS activities in ER/PR(+) subtypes.

Recent proteogenomic studies by the CPTAC have provided MS-based quantitation of proteins expressed in various breast cancer subtypes (64, 65). The data mining studies of these proteomics data repositories allowed us to compare the expression of the same nuclear- and mt-encoded OXPHOS subunits in breast cancer luminal A and B subtypes which mainly correspond to the ER/PR(+) subtype that we analyzed in this study. Additionally, the expression of nuclear-encoded subunits SDHA, ATP5A1, UCQRC2, and SDHB displayed more variability while the mt-encoded subunit MT-COII and NDUFB8 expressions were lower in more than half of the tumor tissues analyzed by the CPTAC (**Figure 1D** and **Table S2**). The agreement between the data from the breast cancer CPTAC proteomics analyses and our immunoblotting studies imply that the reduced steady-state expression of complex I and IV subunits, NDUFB8 and MT-COII, respectively, contributes to the heterogeneity of energy metabolism in ER/PR(+) breast cancer.

### Modulation of OXPHOS by mitochondrial translation in ER/PR(+) breast cancer

**Figure 2:**
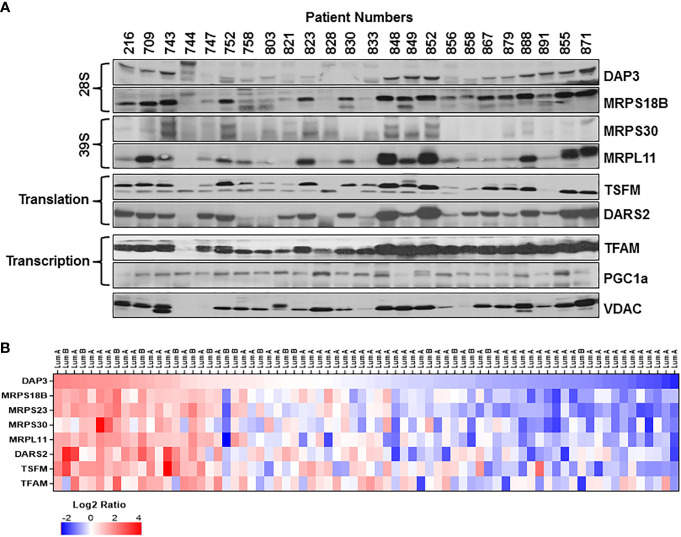
Mitochondrial translation and transcription related protein expression in ER/PR(+) breast cancer. **(A)** Relative expression of the mitochondrial small (28S) and large (39S) subunit proteins DAP3, MRPS18B, MRPS30, and MRPL11 were detected by immunoblotting analyses. TSFM and DARS2 are the two other mitochondrial translation related factors. Mitochondrial transcription related proteins, TFAM and PGC1a, as well as VDAC are also detected by immunoblotting of tumor lysates. Approximately 20 μg of lysates were separated by 12% SDS-PAGE and equal protein loading was evaluated by probing membranes with Ponceau S staining and GAPDH (data not shown). **(B)** Expression of various MRPs in the CPTAC data (65) set as described in **Figure 1D** legend. DAP3 expression in luminal A and B breast tumors is ranked from high (red) to low(blue) expression to show the correlation between the MRPs and the translation related factors DARS2 and TSFM.

Based on our studies of mitochondrial translation over the decades, one of the most obvious causes for the reduced expression of mt-encoded subunits of complex IV such as MT-COI and MT-COII is a defect in the translation machinery including ribosomes and translation factors. For this reason, we carried out immunoblotting analyses of several MRPs associated with breast cancer as well as mitochondrial translation and transcription factors in tumor biopsies for their impacts on OXPHOS subunit expression. As shown in **Figure 2A**, the changes in the small (28S) and the large (39S) ribosomal subunit proteins, DAP3 (MRPS29), MRPS18B, MRPS30, and MRPL11, reflected the changes observed in MT-COII and NDUFB8 expressions obtained for the same patient (**Figures 1A**, **2A**). The other translation-related proteins mitochondrial translation elongation factor Ts (TSFM) and mitochondrial aspartyl-tRNA synthetase (DARS2) also followed a relatively similar pattern to MRP, MT-COII, and NDUFB8 expressions when compared to the same patient. This observation allowed us to correlate MRP expressions to OXPHOS subunit expression and group their expressions as high, medium, and low in 26 ER/PR(+) biopsies (**Figures 1B**, **2B** and **Supplementary Figure 3**). On the other hand, neither nuclear and mitochondrial transcription factors, PGC1a and TFAM, respectively, nor VDAC and CS protein expressions could be correlated to OXPHOS subunit expression in tumor biopsies (**Figure 2A**). Therefore, this led us to suggest that mitochondrial translation is one of the major determinants of the energy metabolism in a significant number of ER/PR(+) biopsies analyzed by immunoblotting in our studies.

The correlation observed between the MRP and mitochondrial translation factor expressions in ER/PR(+) tumor biopsies was also assessed using the CPTAC breast cancer proteomics data published by Krug et al. (65). The heat map analysis of MRPs and various mitochondrial translation-related proteins factors also verified the reduced expression of these proteins in the majority of the ER/PR(+) luminal A and B subtypes (**Figure 2B** and **Table S2**). Moreover, the majority of the small and large subunit MRP expressions followed a consistent pattern patient to patient in all the breast cancer subtypes (**Figure S4**). Another significant observation was the reduced expression of MRPs seen in over 70% of the ER/PR (+) luminal A subtype. The CPTAC proteomics data and our results suggest that the MRP and/or mitochondrial translation-related protein expression can be used as prognostic markers of tumor metabolism in ER/PR(+) breast cancer and its subtypes.

### Expression of epithelial-mesenchymal transition markers and metalloprotease-2 in ER/PR(+) breast cancer

Epithelial to mesenchymal transition (EMT) is associated with metastatic characteristics of tumors as well as their resistance to therapy. In many cases, this transition occurs through a hybrid state identified by the expression of many different markers for the epithelial, hybrid, or mesenchymal states (72). The best predictors for these states are determined by vimentin (VIM) and E-cadherin (CHD1) gene expression ratio combined with claudin 7 (CLDN7) expression using the TCGA breast cancer datasets (73) as well as the correlation of MMP2 expression to the progression of breast cancer to bone metastasis (74). Overexpression of the two mitochondrial proteins, citrate synthase (CS) and complex V subunit, ATP5C1 (or ATP5F1C), are described as markers for aggressiveness in triple negative breast cancer (75, 76).

**Figure 3:**
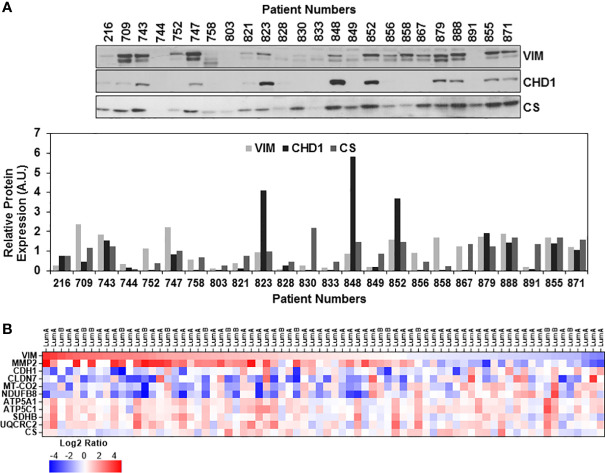
Expression of epithelial-mesenchymal transition markers in ER/PR(+) breast cancer. **(A)** Expressions of vimentin (VIM), E-cadherin (CHD1), and citrate synthase (CS) were detected by immunoblotting using 20 μg of patient biopsies as described in **Figures 1A** and **2A**. Relative VIM, CHD1, and CS expressions were quantified by normalizing antibody signal intensities to protein loading. **(B)** Expression of VIM, metalloprotease-2 (MMP2), CHD1, and claudin-7 (CLDN7) was compared to MT-COII and NDUFB8 log2 expression ratios to demonstrate the changes in EMT and metastatic markers using the CPTAC data set (65) (see text for the discussion). VIM expression is ranked from high (red) to low (blue). Increased VIM and MMP2 expression in majority of the luminal A and B biopsies correspond to the decreased OXPHOS subunit expression (MT-COII and NDUFB8) and epithelial markers (CHD1 and CLDN-7).

The EMT status of the ER/PR(+) breast tumor biopsies was evaluated by immunoblotting analyses of VIM, CHD1, and CS expressions. As anticipated, the majority of the biopsies expressed high levels of VIM while half of the samples expressed both VIM and CHD1 (**Figure 3A**). Expression of both VIM and CHD1 is interpreted as the hybrid state as often observed in the TCGA breast tumor data sets (73). There was no correlation between the tumor stages and high VIM and CS expressions; however, some of the biopsies still preserved the CHD1 expression (**Figure 3A**). When the expression of EMT markers was compared to OXPHOS subunit expression for the same patient, the patients with high to moderate OXPHOS subunit expression, specifically the MT-COII, with the CHD1 expression suggesting a hybrid EMT state for the patient (**Figure 3A**).

Although immunoblotting analyses were not directly comparable due to different signal intensities, ER/PR(+) breast tumor proteomics data allowed us to correlate mitochondrial energy metabolism and translation to EMT status further. The log2 protein expression values for OXPHOS subunits and mitochondrial translation components used in **Figures 1D**, **2B**, respectively, were compared to VIM, CLDN7, and MMP2 expression going from high to low VIM expression (**Figure 3B** and **Table S2**). The VIM and MMP2 expression trends were in agreement while CLDN7 expression increased as VIM and MMP2 decreased. In the majority of the luminal A and B biopsies with high VIM and MMP2 expression, both MT-COII and NDUFB8 decreased (**Figure 3B**). This tendency was also observed in mitochondrial translation components (**Figure S5**). As seen in immunoblotting analyses of ER/PR(+) biopsies, the OXPHOS subunits, specifically the MT-COII and NDUFB8, expressions were proportional to the changes in mitochondrial translation components rather than the mitochondrial transcription related factors TFAM and PGC1a (**Figures 1A**, **2A**). Moreover, other mitochondrial proteins involved in breast cancer aggressiveness, such as ATP5C1 and CS did not show any specific tendency or change with increased VIM and MMP2 expressions in luminal A and B breast cancer subtypes (**Figure 3B**). Therefore, the modulation of mitochondrial translation and/or expression of its components could be significant in remodeling of energy metabolism and EMT transition in ER/PR (+) breast cancer.

### Defects in OXPHOS and mitochondrial translation in breast cancer cell lines

**Figure 4:**
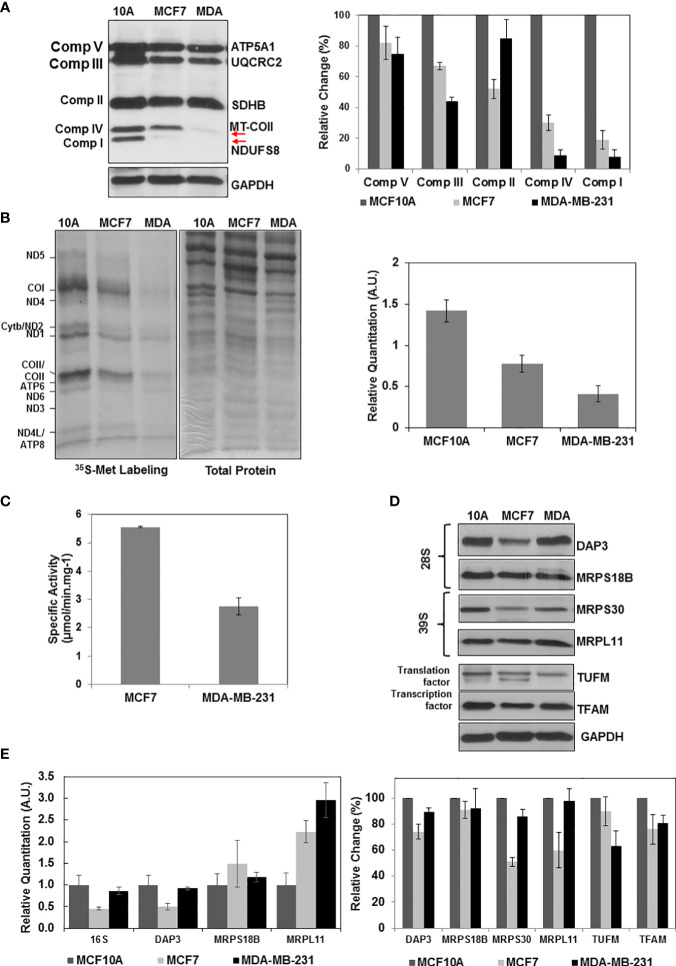
Altered OXPHOS subunit expression and mitochondrial translation in breast cancer cell lines. **(A)** The OXPHOS subunit expression was detected by immunoblotting of approximately 30 μg lysates obtained from breast cancer cell lines MCF10A (10A), MCF7 (MCF7), and MDA-MB-23 (MDA) as described in **Figure 1A**. The relative quantitation of OXPHOS subunit expression represent the mean ± SD of at least three experiments with MCF10A subunit expression adjusted to 100%. **(B)** Mitochondrial translation is determined by ^35^S-Met pulse labeling of 13 mt-encoded OXPHOS subunits in breast cancer cell lines. The 13 mt-encoded complex I subunits ND1-ND6; complex III subunit cyt b; complex IV subunits COI-COIII; and complex V subunits ATP6 and ATP8 are labeled on the autoradiography of the 13% SDS-PAGE. Total protein loading (30 μg) is visualized by Coomassie Blue staining of the 13% gel. Relative quantitation of mitochondrial translation in MCF10A, MCF7, and MDA-MB-231 cells represents the mean ± SD of the pulse labeling of 13 mt-encoded proteins. **(C)** The complex IV specific activity was determined using (~20 μg) of MCF7 and MDA-MB-231 cell lysates as described in **Figure 1C**. **(D)** Immunoblotting analyses of MRPs (DAP3, MRPS18B, MRPS30, and MRPL11) and mitochondrial translation (TUFM) and transcription (TFAM) related proteins in breast cancer cell lines. Equal protein loading was determined by probing membranes with GAPDH and Ponceau S staining (data not shown). Results represent the mean ± SD of at least three experiments. **(E)** Relative changes in mt-encoded ribosomal large subunit rRNA, 16S rRNA, and the MRP mRNAs, DAP3, MRPS18B, and MRPL11 were determined by quantitative RT-PCR in the MCF10A, MCF7, and MDA-MB-231 cell lines. Results represent the mean ± SD of at least three experiments adjusted to MCF10A mRNA expression as the 100%.

Previously, mtDNA mutations, low copy numbers, and reduced mitochondrial transcript levels were associated with increased metastasis and poor prognosis in breast cancer (6–9). The results presented in **Figures 1**, **2** also clearly demonstrated that the ER/PR(+) breast tumor biopsies have a high degree of OXPHOS heterogeneity supported by the changes in MRP expression or reduced protein synthesis in mitochondria. The majority of the biopsies had medium or low OXPHOS levels, while OXPHOS and MRP expression levels were significantly increased in the remainder. It is then highly likely that the low OXPHOS levels are due to the defects in mitochondrial biogenesis including mitochondrial translation and transcription in the ER/PR(+) biopsies. To recapitulate these observations, we first determined the OXPHOS subunit expression in a non-tumorigenic epithelial breast cell line MCF10A, ER/PR(+) MCF7, and triple-negative MDA-MB-231 cell lines by immunoblotting (**Figure 4A**). Similar to the patient biopsies, complex I and IV subunit expressions were more variable than the complex II, III, and V subunits specifically in MCF7 and MDA-MB-231 cell lines (shown by arrows in **Figure 4A**). The variation at the steady-state nuclear and mitochondrial-encoded protein OXPHOS subunit expression was also reflected in OXPHOS activities in these cell lines (4).

We next investigated the role of mitochondrial translation on reduced OXPHOS subunit expression and complex IV activities in breast cancer cell lines. For this purpose, [^35^S]-Met pulse-labeling of *de novo* synthesized mt-encoded proteins was performed in the presence of emetine, an inhibitor of cytosolic protein synthesis (66). In this analysis, [^35^S]-Met is only incorporated into the 13 mt-encoded OXPHOS subunits by the mitochondrial translation machinery (**Figure 4B**). The *de novo* protein synthesis relative to that of the MCF10A non-tumorigenic epithelial breast cell line was reduced by about 50% and 70% in MCF7 and MDA-MB-231 cell lines, respectively (**Figures 4B**). The effect of reduced OXPHOS subunit expression shown in **Figures 4A, B** also was observed in complex IV assays performed using MCF7 and MDA-MB-231 cell lysates. The *de novo* mitochondrial protein synthesis in MDA-MB-231 cells was approximately 50% of the protein synthesis in MCF7 cells (**Figure 4B**). This reduction was in agreement with the complex IV activities determined in MCF7 and MDA-MB-231 cells (**Figure 4C**).

The defect observed in mitochondrial translation could be due to defect(s) in the expression of mitochondrial translation components or their transcription. In order to assess their effect on OXPHOS subunit expression, we performed immunoblotting and qRT-PCR analyses in these cell lines. Quantitation of immunoblots showed a clear reduction in several MRPs, such as DAP3 and MRPS30, as well as mitochondrial transcription factor TFAM in ER/PR(+) MCF7 cell lines (**Figure 4D**). A clear reduction at the steady-state expression of MRPs was not observed in our analyses of MDA-MB-231 cells; however, decreased expression of MRPS23, one of the 30 mitochondrial small subunit ribosomal proteins, and as well as its methylation at Arg and Lys residues have shown to inhibit OXPHOS and promote the aggressive and metastatic types of breast cancer (61). At the transcription level, 16S rRNA and DAP3 transcripts were reduced in both MCF7 and MDA-MB-231 cell lines (**Figure 4E**). The other MRP transcripts on the other hand did either not significantly change or increased 2-3 folds possibly to compensate for the reduced synthesis of MRPs, TFAM, or TUFM in these cell lines (**Figure 4E**). The 50% decrease observed in *de novo* protein synthesis in MCF7 cell lines could be caused by defects in mitochondrial translation and transcription (**Figure 4B**). The only factor that decreased in the MDA-MB-231 cell line was the mitochondrial translation factor TUFM. The four-fold reduction in mitochondrial translation cannot only be explained by the 35% reduction in TUFM expression in MDA-MB-231 (**Figures 4B, D**). MDA-MB-231 has relatively high mitochondrial heteroplasmy associated with higher invasion and metastatic capabilities supported by the glycolytic energy metabolism rather than OXPHOS compared to the MCF7 cell lines (77). In fact, the MS-based proteomics studies performed in our laboratory clearly show that the expression of glycolytic enzymes increased significantly in MDA-MB-231 cells relative to that of MCF7 cell lines (**Table S3**). The changes determined in the *de novo* protein synthesis and in expression of mitochondrial translation components in these cell lines could also be correlated to alterations observed in 26 ER/PR(+) breast tumor biopsies.

## Conclusions and future directions

Evidence suggests that the mutations and defects in mtDNA and the proteins supporting OXPHOS and apoptosis alter mitochondrial function in disease, specifically in cancer. Undeniably, mitochondria play a key role in these processes; however, we are far from understanding the cellular and molecular mechanisms maintaining this balance. In this study, we investigated the changes in the expression of OXPHOS subunits and MRPs responsible for the synthesis of 13 mt-encoded subunits in 26 ER/PR(+) biopsies. Alterations in the expression of MRPs and mitochondrial translation-related proteins directly influenced the expression of complex I and IV subunits, NDUFB8 and MT-COII, in the majority of the biopsies. The strong agreement between the OXPHOS subunit expression analysis by immunoblotting and the MS-based proteogenomic studies by the CPTAC have provided confidence in our hypothesis given above. Although we were not able to associate tumor stages and OXPHOS subunit expression, the reduced OXPHOS and MRP expression in the majority of the ER/PR(+) biopsies is noteworthy.

With the metabolic heterogeneity of tumor cells in mind, one of the controversies that need to be resolved is the contribution of OXPHOS to aggressiveness and development of chemo-resistance and recurrence in breast cancer. At large, defects and mutations in mtDNA and other OXPHOS components are known to induce invasiveness and recurrence in breast cancer (12, 13, 47). In several histopathological and patient-derived primary cell studies, the increased mitochondrial mass has been shown to develop cancer stem-like cells, which are more chemo-resistant, resulting in tumor recurrence, and distant metastasis (12, 16, 42). In conclusion, further elucidation of mitochondrial transcription and translation machineries supporting OXPHOS is necessary to identify the mechanism(s) fueling breast cancer invasiveness, metastasis, and chemo-resistance.

## Data Availability Statement

The datasets presented in this study can be found in online repositories. The names of the repository/repositories and accession number(s) can be found in the article/**Supplementary Material**.

## Author contributions

EK and HK designed the study. EK, FCK, and FK prepared biopsies and breast cancer cell lines for immunoblotting analyses and activity assays. EK performed ^35^S-Met pulse labeling assays. FCK and EK performed the data mining analysis of the CPTAC proteome and prepared the figures. EK, MT, and HK involved in writing or revising the manuscript and the grant proposal that partially funded this work. All authors contributed to the article and approved the submitted version.

## Funding

This research was partially funded by Edwards Comprehensive Cancer Center to EK, HK, and MT and The Scientific and Technological Research Council (TUBITAK) of Turkey to FK. The tissue procurement center at the Marshall University Edwards Comprehensive Cancer Center was supported by a grant (2U54GM104942) from the National Institute for General Medical Sciences (NIGMS) awarded to the West Virginia Clinical and Translational Science Institute (WV-CTSI).

## Acknowledgments

The authors would like to thank Tissue Procurement Center at the Edwards Comprehensive Cancer Center at the Marshall University for providing tumor and normal breast biopsies. We also gratefully acknowledge Biomedical Sciences Department at Joan C. Edwards School of Medicine, Marshall University for its support. The authors also appreciate the discussions regarding data analysis with Drs. Vincent Sollars and James Denvir.

## Conflict of interest

The authors declare that the research was conducted in the absence of any commercial or financial relationships that could be construed as a potential conflict of interest.

## Publisher’s note

All claims expressed in this article are solely those of the authors and do not necessarily represent those of their affiliated organizations, or those of the publisher, the editors and the reviewers. Any product that may be evaluated in this article, or claim that may be made by its manufacturer, is not guaranteed or endorsed by the publisher.

## Supplementary material

Data Sheet 1 - Supplementary Figures.
