# Supplementary material for: Role of mitochondrial translation in remodeling of energy metabolism in ER/PR(+) breast cancer
Source: Front Oncol. 2022 Aug 30;12:897207. doi: 10.3389/fonc.2022.897207 (PMC9472243; doi:10.3389/fonc.2022.897207)
Supplement: Supplementary file 1 [file DataSheet_1.pdf]

SUPPLEMENTAL FIGURES

Figure S1. Immunoblotting analysis of OXPHOS subunits in ER/PR(+) normal and tumor breast tissue lysates. The expression of OXPHOS subunits, including NDUF8 (complex I), SDHA (complex II), UQCRC2 (complex III), COII (complex IV), and ATP5A (complex V) were detected by immunoblotting analyses. Approximately, 20 µg of lysate from each patient's normal and tumor biopsies were separated on 12% SDS-PAGE, and equal protein loading was normalized to GAPDH antibody signal and Ponceau S staining of the membranes. As shown by the arrow, albumin (ALB) protein expression largely varied between the adjacent normal and tumor tissues obtained from the same patient.

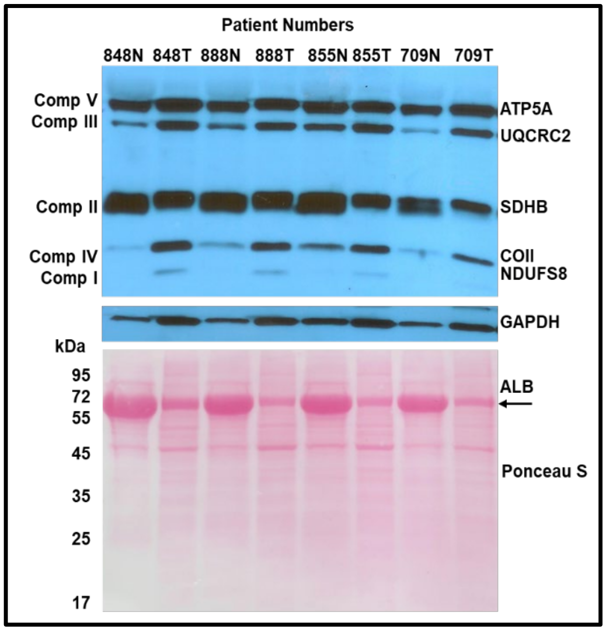

Figure S2. Ponceau S staining of the membrane used in Fig. 1 to detect OXPHOS subunits in ER/PR(+) tumor tissue lysates. Approximately, 20 µg of protein lysates obtained from ER/PR (+) tumor tissues was separated by 12% SDS-PAGE, and equal protein loading was evaluated by GAPDH antibody (see Fig 1A) and Ponceau S staining. Quantitation of OXPHOS subunit expression in patient tumor biopsies was normalized to protein loading determined for this and two other Ponceau S stained gels.

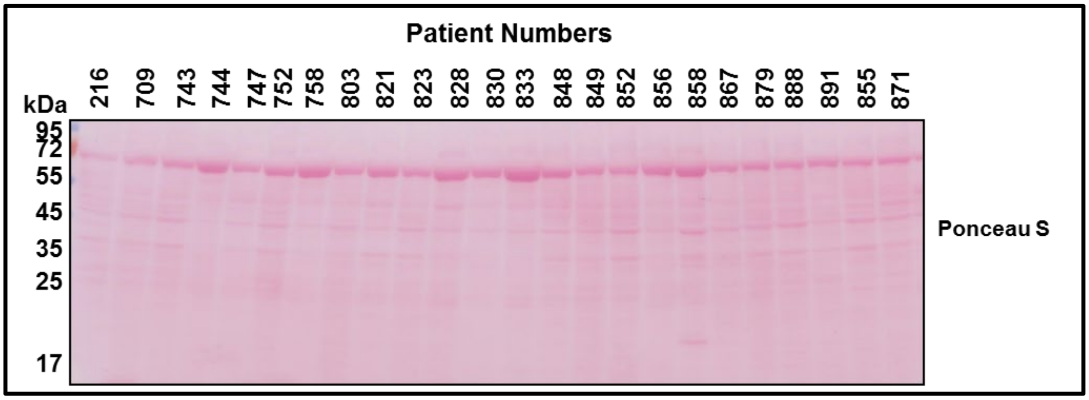

Figure S3. Complex IV activity assays. Complex IV activity assays were performed using several selected tumor tissues using as described in Materials and Methods [1]. To normalize the protein amounts used in complex IV activity assays, approximately 20 µg of normal and tumor tissue lysates were separated on 12% SDS-PAGE and transblotted onto nitrocellulose membranes. Protein bands in Ponceau S-stained membranes were quantified below the albumin (ALB) bands (shown by arrows).

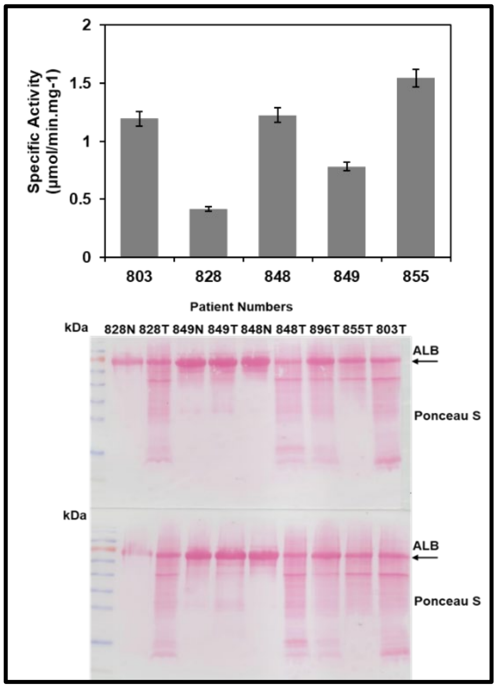

Figure S4. Mitochondrial Ribosomal Protein (MRP) expression in CPTAC breast cancer proteome. Log2 protein expression ratios of MRPs plotted for luminal A, luminal B, basal, and Her2 subtypes of invasive ductal carcinoma. Proteome data is taken from Krug *et* al. presented in Supplemental Table 2 (Excel sheet B)[2].

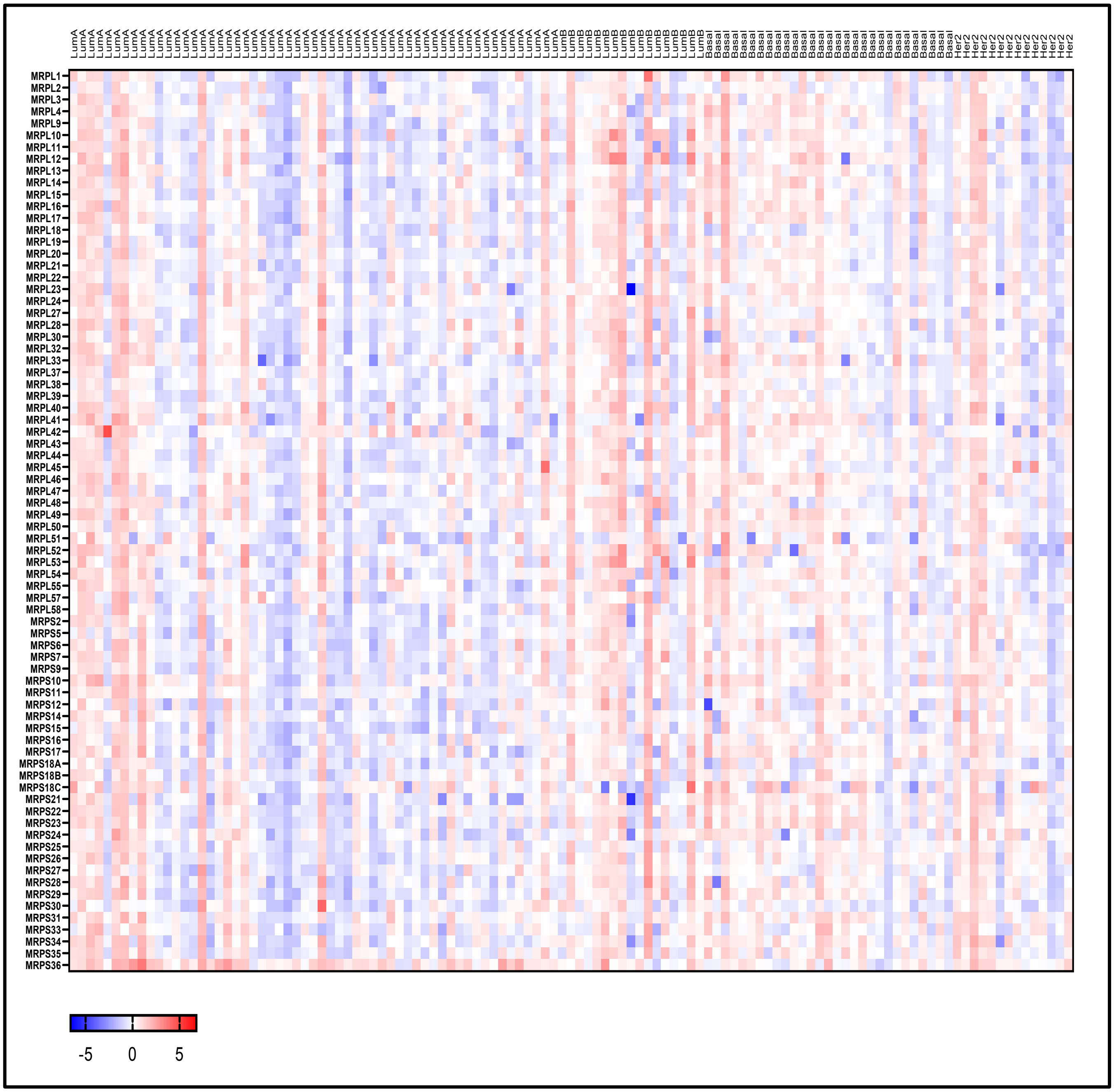

Figure S5. Correlation of EMT markers, OXPHOS subunits, and Mitochondrial Ribosomal Protein Expression in CPTAC breast cancer proteome. Log2 protein expression ratios of VIM, MMP2, MRPs plotted for luminal A and luminal B subtypes of invasive ductal carcinoma. Protein expression was ranked from highest to lowest vimentin (VIM) expression in each patient. Correlation between VIM and metalloprotease-2 (MMP2) expression shows the epithelial-to-mesenchymal transition and metastatic progression of breast tumors. Interestingly, expression of OXPHOS subunits (MT-COII and NDUFB8) and mitochondrial translation related proteins (DAP3, MRPS18B, MRPS23, MRPS30, MRPL11, DARS2, and TSFM) were reduced mostly in patient biopsies with high VIM and MMP2 expressions. Proteome data is taken from Krug et al presented in Table S2 (sheet B)[2].

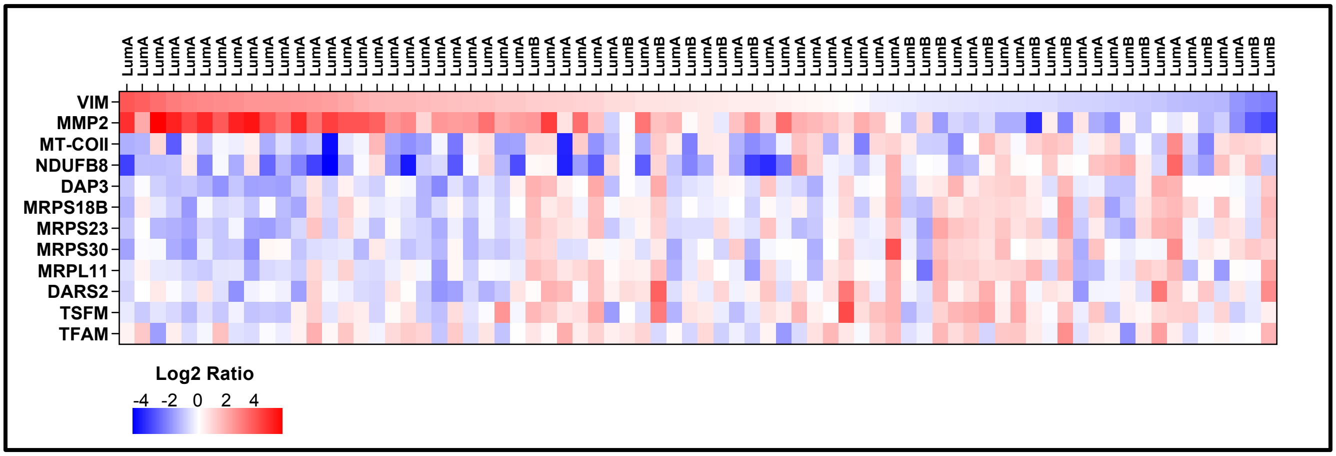

REFERENCES

1. Birch-Machin, M.A. and D.M. Turnbull, *Assaying mitochondrial respiratory complex activity in mitochondria isolated from human cells and tissues.* Methods Cell Biol, 2001. **65**: p. 97-117.
2. Krug, K., et al., *Proteogenomic Landscape of Breast Cancer Tumorigenesis and Targeted Therapy.* Cell, 2020. **183**(5): p. 1436-1456 e31.
