# Supplementary material for: Role of mitochondrial translation in remodeling of energy metabolism in ER/PR(+) breast cancer
Source: Front Oncol. 2022 Aug 30;12:897207. doi: 10.3389/fonc.2022.897207 (PMC9472243; doi:10.3389/fonc.2022.897207)
Supplement: Supplementary file 3 [file Table_1.pdf]

Table S1. The tumor characteristics of 26 ER/PR(+) invasive ductal carcinoma biopsies.

Patient Number	Age	Pathologic Staging	ER	PR	HER-2/neu
216	54	pT3pN1a(sn)	Positive	Negative	Negative
709	56	ypTUncertain	Positive	Positive	Negative
743	63	pT1cpNO(sn)	Positive	Positive	Negative
744	67	pT2pNO(sn)	Positive	Positive	Negative
747	79	pT2pNO(sn)	Positive	Positive	Negative
752	74	pT1cpN1a	Positive	Positive	Negative
758	70	pT2pNO(sn)	Positive	Positive	Negative
803	52	pT3NO	Positive	Positive	Negative
823	50	pT2N1Q	Positive	Positive	Negative
828	76	pT2pNO(sn)	Positive	Positive	Negative
830	42	pT1cpW1mi	Positive	Positive	Negative
833	61	pT3pN2a(sn)	Positive	Positive	Negative
844	59	pT3pNO(sn)	Positive	Positive	Uncertain
848	59	pT2snpNO	Positive	Positive	Negative
849	50	pT1csnpNO	Positive	Positive	Uncertain
852	49	pT2pNO(sn)pM	Positive	Positive	Negative
855	57	ypT2ypN1a	Positive	Positive	Uncertain
856	47	pT1cpNO(sn)pM	Positive	Positive	Uncertain
858	55	pT2snpNO	Positive	Positive	Negative
867	75	pT2snpNO	Positive	Positive	Negative
871	63	ypT2snpNO	Positive	Negative	Negative
873	40	pT2snpNO	Positive	Positive	Negative
879	65	pT1cpNO(sn)	Positive	Positive	Uncertain
888	59	pT2pNO(sn)pM	Positive	Positive	Uncertain
891	59	pT2pNapM	Positive	Positive	Negative
893	60	ypT2pN1apM	Positive	Negative	Negative

pT: Tumor stage
pN: Lymph node metastasis
pM: Distant metastasis
