# Supplementary material for: Role of mitochondrial translation in remodeling of energy metabolism in ER/PR(+) breast cancer
Source: Front Oncol. 2022 Aug 30;12:897207. doi: 10.3389/fonc.2022.897207 (PMC9472243; doi:10.3389/fonc.2022.897207)
Supplement: Supplementary file 5 [file Table_3.pdf]

Table S3. Expression of glycolytic enzymes detected by LC-MS/MS analyses of MCF7 and MDA-MB-231 protein lysates.

Gene Name	Protein Name	MW (Da)	MCF7 (Heavy)	MDA-MB-231 (Light)	MCF7/MDA-MB-231 Ratio
ALDOA	Fructose-bisphosphate aldolase A	39,420	46	121	0.38
ENO1	Alpha-enolase	47,168	69	216	0.32
GAPDH	Glyceraldehyde-3-phosphate dehydrogenase	36,053	314	406	0.77
G6PI	Glucose-6-phosphate isomerase	63,146	62	36	1.72
PKM	Pyruvate kinase	57,936	120	389	0.31
LDHA	L-Lactate dehydrogenase A	36,688	13	80	0.16
LDHB	L-Lactate dehydrogenase B	36,638	1	44	0.02
PFKM	ATP-dependent 6-phosphofructokinase	85,595	23	25	0.92
PGAM1	Phosphoglycerate mutase 1	28,804	14	49	0.29
PGK1	Phosphoglycerate kinase 1	44,796	13	29	0.45
PGM1	Phosphoglucomutase-1	61,448	3	18	0.17
TPIS	Triosephosphate isomerase	30,791	31	49	0.63

Mass-spectrometry (MS)-based identification of glycolytic enzymes in MCF7 and MDA-MB-231 cell lines using **stable isotope labeling of amino acids in cell culture (SILAC)** in combination with LC-MS/MS analysis using protocols provided by the manufacturer (Thermo Fisher Scientific, Inc.). In this approach, MCF7 cell lines were grown in DMEM media containing stable isotope labeled (heavy) amino acids $^{13}C_6$-Lys (K6) and $^{13}C_6$-Arg (R6) while the MDA-MB-231 cells were grown in media containing regular (light) amino acids ($^{12}C_6$-Lys (K0) and $^{12}C_6$-Arg (R0)). Equal amounts of cell lysates obtained from heavy- and light-labeled, MCF7 and MDA-MB 231, respectively, were combined and separated on a 14% SDS-PAGE. The protein lanes containing MCF7 and MDA-MB-231 were divided into ~1 mm pieces and subjected to trypsin digestion. The tryptic digests were analyzed by LC-MS/MS, which consisted of a Surveyor auto sampler and HPLC and an LTQ linear ion trap mass spectrometer (Thermo Fisher Scientific, Inc.). Protein identification was performed by Mascot Distiller (Matrix Science). The number of heavy- and light-labeled peptides of glycolytic enzymes detected by LC-MS/MS is directly proportional to protein expression in these cell lines.
